# Does Benchmarking of Rating Scales Improve Ratings of Search Performance Given by Specialist Search Dog Handlers?

**DOI:** 10.3389/fvets.2021.545398

**Published:** 2021-02-02

**Authors:** Corinna C. A. Clark, Nicola J. Rooney

**Affiliations:** ^1^Department of Life Sciences, University of Warwick, Coventry, United Kingdom; ^2^Animal Welfare and Behavior Group, Bristol Veterinary School, University of Bristol, Bristol, United Kingdom

**Keywords:** search dog, detection dog, rating scale, performance, measurement, scale design, quantification

## Abstract

Rating scales are widely used to rate working dog behavior and performance. Whilst behaviour scales have been extensively validated, instruments used to rate ability have usually been designed by training and practitioner organizations, and often little consideration has been given to how seemingly insignificant aspects of the scale design might alter the validity of the results obtained. Here we illustrate how manipulating one aspect of rating scale design, the provision of verbal benchmarks or labels (as opposed to just a numerical scale), can affect the ability of observers to distinguish between differing levels of search dog performance in an operational environment. Previous studies have found evidence for range restriction (using only part of the scale) in raters' use of the scales and variability between raters in their understanding of the traits used to measures performance. As provision of verbal benchmarks has been shown to help raters in a variety of disciplines to select appropriate scale categories (or scores), it may be predicted that inclusion of verbal benchmarks will bring raters' conceptualization of the traits closer together, increasing agreement between raters, as well as improving the ability of observers to distinguish between differing levels of search dog performance and reduce range restriction. To test the value of verbal benchmarking we compared inter-rater reliability, raters' ability to discriminate between different levels of search dog performance, and their use of the whole scale before and after being presented with benchmarked scales for the same traits. Raters scored the performance of two separate types of explosives search dog (High Assurance Search (HAS) and Vehicle Search (VS) dogs), from short (~30 s) video clips, using 11 previously validated traits. Taking each trait in turn, for the first five clips raters were asked to give a score from 1, representing the lowest amount of the trait evident to 5, representing the highest. Raters were given a list of adjective-based benchmarks (e.g., very low, low, intermediate, high, very high) and scored a further five clips for each trait. For certain traits, the reliability of scoring improved when benchmarks were provided (e.g., Motivation and Independence), indicating that their inclusion may potentially reduce ambivalence in scoring, ambiguity of meanings, and cognitive difficulty for raters. However, this effect was not universal, with the ratings of some traits remaining unchanged (e.g., Control), or even reducing in reliability (e.g., Distraction). There were also some differences between VS and HAS (e.g., Confidence reliability increased for VS raters and decreased for HAS raters). There were few improvements in the spread of scores across the range, but some indication of more favorable scoring. This was a small study of operational handlers and trainers utilizing training video footage from realistic operational environments, and there are potential cofounding effects. We discuss possible causal factors, including issues specific to raters and possible deficiencies in the chosen benchmarks, and suggest ways to further improve the effectiveness of rating scales. This study illustrates why it is vitally important to validate all aspects of rating scale design, even if they may seem inconsequential, as relatively small changes to the amount and type of information provided to raters can have both positive and negative impacts on the data obtained.

## Introduction

Rating scales are used across numerous fields to assess differences between individuals (human and animal), e.g., in the occurrence of particular behaviors or medical conditions (1, 2), the degree of pain experienced (or inferred in the case of animals) (3, 4), mood and quality of life (5–7), marketing preferences (8, 9), as well as being widely used to assess performance in specific tasks or roles (10, 11). They are used widely when quantifying the performance of working dogs both in selection tests [e.g., (12–14)] and in their working role [e.g., (15, 16)].

Search, or detection, dogs are used for many purposes, for example: to locate target species in wildlife conservation (17); in human medicine, to identify patients with cancer (18); or to assist people with medical conditions (19–21); and by various law enforcement agencies to find people, drugs, money and explosives [e.g., (15, 22, 23)]. Monitoring of search dog performance is essential to maintain the effectiveness of individual dog-handler teams by highlighting any short-term training needs, but it is also critical to direct longer term strategies for improving ability of working dogs. To ensure that ratings provide accurate and reliable information it is important that any performance measurement tool is designed appropriately, with relevant and quantifiable measures, which accurately reflect differences between the subjects being rated. Irrespective of the purpose there are two elements to the rating process, and therefore two main potential sources of error or variance: the design of the measurement tool (e.g., rating scale, questionnaire or survey), and factors associated with the observer/rater. Elsewhere we deal with the latter (24), here we are primarily interested in the former, the measurement tool, and how manipulating specific aspects of rating scale design can affect the ability of observers (in this case dog handlers) to distinguish between differing levels of performance.

There is a growing body of research exploring and validating scales for rating dog behavior during temperament and behavior tests. Researchers have systematically examined how best to quantify dog behaviors [e.g., (14, 25–27)], demonstrating, for example, that rating scales used by trained observers (14), or researchers (28) provide ratings similar to those made by working dog experts and that scales are as successful as behavioral coding in predicting which dogs would be successfully selected as odor detection dogs at 12 months of age (27). However, when investigating which factors affect and best predict working ability, behavioral measures are usually compared against training or practitioners' organizations' own measures of success. Whilst some studies explore predictors of successful acceptance into training (27), other explore predictors of successful certification (23), binary outcomes, which although practically very important, lack granulation. Other studies rely on scales devised by the working dog organization, such as those used in competitive hunting trials (16), which were often formed historically without scientific input and without thorough consideration of how their design may influence potential sources of error and the quality of information obtained. There is considerable evidence that seemingly small changes in scale design can alter the way raters interpret and use scales, therefore affecting the reliability and validity of data (29, 30). Application and investigation of these principles when rating working dog performance will allow us to devise meaningful scales for investigating factors impacting performance.

In Rooney and Clark (31), we detailed a systematic process of selecting and testing suitable behavioral trait measures (e.g., Motivation to search, Confidence in the environment) for dogs trained to search for explosives on/in vehicles (VS dogs), and high assurance search (HAS) dogs, trained to detect buried improvised explosive devices (IEDs). These instruments were designed as a method of recording day-to-day variation in performance, using the most appropriate traits for each search classification. We found good reliability for assessing dog performance within a group of raters, but it would appear that some raters in the group were better able to use the 1–5 scales reliably than others and the predicted reliability if a single rater were to provide scores was poor. Therefore, we could not be confident that individual handlers could provide comparable data. As handlers often work alone, it is important to make the measures practically applicable and viable for a single a handler to use and our aim here was to find a method of improving the use of the scales at the individual level and increase single rater reliability to an acceptable threshold.

One plausible reason for low single rater reliability (31) was that the raters may not have agreed on, or even understood some of the measurement traits, making it difficult to reliably categorize the behavior they observed into points on a 1–5 scale. Where raters have difficulty in conceptualizing an aspect of performance they may resort to careless rating, bias (e.g., halo or leniency), or using a restricted range of values on the scale - typically mid-range to positive range (net acquiescence) or at the extremes (8, 32). Our previous study of handler ratings found evidence for range restriction in the use of the scales (24, 31), which could reduce accuracy (real performance compared to scores), agreement between raters, and the ability to distinguish between performances - as ratees are, in effect, being scored on much smaller scales than intended. In order for ratings to be reliable, meanings of traits and any other performance measures must therefore be clear.

How raters encode, organize, integrate, recall and evaluate information involves an “on-line” (33) or internal evaluation, where categorization or judgements are made, based on raters' own idiosyncratic understanding of a trait or concept (34). To assign a performance score, behaviors which generally occur on a continuum are assessed according to which of several discrete (i.e., non-continuous) categories they most closely match. To maximize the accuracy of this categorization it is therefore important to bring each rater's idiosyncratic categorization (based on their own internal mental representation) closer to a common understanding of which behaviors constitute specific levels of each dimension of performance. If this can be achieved then we would expect raters shown examples of behavior at each level of performance to be able to utilize the full range of the scale.

Providing verbal benchmarks (anchors or labels) as opposed to just providing a numerical scale, has been shown to help raters to select an appropriate category, increasing reliability and validity [e.g., (35, 36)]. These verbal anchors can be single words or short descriptions and are generally “adjective-based” (good, poor, high, low, average) or “descriptive,” providing details about the construct and what each level of performance means (37). Adding descriptive anchors for each level of the behavioral measure should help to clarify the meaning of traits, removing an interpretive step where meanings could be confused between raters, also reducing the cognitive burden of the rater in interpreting their internal mental representation into a point on a scale (38). The use of verbal anchors has been shown to alter the way in which raters use scales (39) and has been recommended to improve agreement between raters (40) and reduce rater bias (32). Descriptive anchors in particular, may be effective at preventing leniency error (37), which is commonly reported in the literature [e.g., (10)] and has also been found to occur when dog handlers rate their own dogs (24). However, the selection of appropriate benchmarks requires careful consideration as providing insufficient detail, too much detail, or altering scales so that they that become emotionally valenced (e.g., too critical) can in fact be detrimental to accuracy and discrimination between ratees (37, 39, 41). In Rooney and Clark (31) raters were asked to apply identical, basic (one or two word) adjective-based descriptors (very low, low, intermediate, high, and very high) for every behavioral measure. The lack of rater reliability may have been a consequence of providing inadequate benchmarks to provide raters with a common concept of each level of performance. This may be a particular issue for behavioral traits that are harder to conceptualize, or where raters are likely to disagree with each other in their meaning. The next stage in developing a dog performance rating tools was therefore to explore the value of providing observers with more detailed verbal benchmarks for each of the levels within every behavioral performance measure, as a method of potentially bringing rater categorizations of performance closer together.

There are of course other considerations and aspects of scale design which can be affected by the addition of benchmarks. These include, the number of scale points, whether some or all points on the scale are benchmarked, and also whether scales are balanced (where the midpoint of the scale equates to the conceptual midpoint). We previously decided that 5-point scales would provide a reasonable trade-off between obtaining enough information and the practicalities of field-based assessment (31). Numerous studies have reported that using scales of typically 5 or 7 points minimizes variability in scale use, maximizes inter and intra test reliability, as well as optimizing cognitive comprehension by raters (41–46). We chose to label all points on the scale, as this is generally considered better than benchmarking only selected points such as the extremes (41, 45). It is also practically feasible to benchmark scales of this size, whereas benchmarking larger scales (particularly if all points are labeled) will increase cognitive burden on the rater (29) and may lead to reduced accuracy due to rating fatigue. Unbalanced scales, where the midpoint of the scale does not equate to the conceptual midpoint of the level of performance, may be useful in discriminating between ratees in a negatively skewed population (47), i.e., where none of the ratees is expected to score at the lowest extremes. But this was not relevant here, as we required discrimination across the full range of the scale.

Although benchmarking is often used in rating scales, including those used on working dogs its efficacy is rarely assessed. Here, we tested the value of providing benchmarks to performance rating scales for two types of explosives search dog. Our aim was to test if, by providing observers with benchmarks for every level (1–5 scale) of search performance, we can: [1] bring raters' conceptualization of the traits closer together, as evidenced by an increase in inter-rater reliability; and [2], increase the ability of raters to discriminate between levels within performance measures, reducing the effect of rating range restriction as measured by a greater use of the 1–5 scale (increased standard deviation). Our observers rated the performance of VS and HAS dogs in training searches using 11 performance measures derived previously (31). Previous work showed that raters of VS dogs assigned differing importance to each of the 11 traits, as well as showing differing levels of reliability for each trait, compared to a raters of HAS dogs. Thus, the impact of benchmarking is likely to differ with the type of search dog being rated. We compared ratings for 10 videoed searches per trait, selected to show as wide a range of performance as possible. The first five for each trait were rated without benchmarks and the second set of five were rated with benchmarks. This was repeated for all 11 traits, therefore each group of raters (VS and HAS) watched and scored 110 video clips in total. This was an opportune study conducted on experienced dog handlers observing dogs in operational environments as part of their own training. It was therefore not possible to randomize the order of video presentation.

## Methods

### Behavioral Measures of Performance

The behavioral measures had been obtained by a systematic process of scale derivation, involving detailed interviews and questionnaires with stakeholders (e.g., trainers, handlers, senior staff) [see (31)]. From this, 12 behavioral trait measures were derived, we selected 11 of these, which could also be scored from short videoed searches on a 1–5 scale (Table 1). Consistency in searching behavior was not included as it could only be assessed from whole searches, not short clips. We did not include the composite measure “Overall Performance” for the same reason, and also because it does not represent a single independent dimension of performance.

**Table 1:** Behavioral measures of performance (traits) in the order they were scored.

**Behavior measures**	
**Short title**	**Full title and description of behavioral trait**
**Control**	Control (responsiveness to verbal and or physical commands). The proportion of commands obeyed and speed of response.
**Motivation**	Motivation (enthusiasm to search). How keen or eager the dog is to search – assessed from the dog's behavior leading up to and at the start of the search.
**Distraction**	Distraction when searching. A distraction is anything that takes the dog's attention away from searching or from starting to search, including urinating.
**Search pattern**	Ability to follow search pattern. How well the dog follows the correct search pattern, without missing areas or needing constant correction. Not following search pattern would include: HAS, pulling off-line, wide back-seek, or following visual cues; VS, pulling/moving away from vehicle being searched, searching ground, or not searching “overlap.”
**Stamina**	Stamina throughout search. How much motivation or enthusiasm decreases over the search, e.g., due to tiredness or loss of confidence.
**Indication**	Strength of indication.
**Confidence**	Confidence (absence of fear/anxiety) How confident or relaxed the dog is.
**Thoroughness**	Thoroughness of search. How much of the search the dog is actively searching: HAS, sniffing with its head down and nose to the ground for the entire search, including on the back-seek and searching right up to the handler; VS, sniffing with nose to the vehicle.
**Independence**	Independence. Ability of the dog to search without guidance, (not needing, or looking for, constant guidance), including being able to continue searching when further away from handler and on back-seek.
**Speed**	Speed of search
**Detect & locate**	Ability to detect and locate scent to source

*Full titles and descriptions are as presented to the raters, but for the sake of brevity the behaviors are referred to in the text by their shortened title (in bold)*.

### Search Videos

Video recordings were made (using Sony Handycam DCR-SR58) of 200 training and accreditation searches (117 VS, 91 HAS), performed by 62 different dogs (35 VS, 27 HAS) in 100 different handler-dog pairings (50 VS; 50 HAS). The same videos had been used to make 17 5 min clips to develop behavioral scales in Rooney and Clark (31); although to avoid repetition different searches or sections within searches were used wherever possible. For each of the 11 behavioral traits we extracted 10 short video clips (each ~30 s), with the aim of illustrating each point on the 1–5 scale, or as wide a range of performance as possible (110 short clips in total). These clips were to be used in a training resource for military dog handling personnel ahead of overseas placement. Both authors viewed and rated the clips and based on their assessments, videos showing a range of ratings were balanced across the pre and post benchmark conditions, with the order shown in the particular set of clips randomized by performance level (i.e., 1, 2, 3, 4, 5).

### Raters

The majority of raters were military (or ex-military) personnel, with the exception of two raters per group who were civilian trainers working within a military establishment. Many of the raters had experience of both VS and HAS, but individuals were assigned to either classification observation group, with only one person appearing in both groups. Raters were all experienced in the classification being studied (16 VS, mean experience with VS 3.2 years, 11 HAS, mean experience 2.3 years) as either dog trainers, course instructors (training search-dog handlers), or as dog handlers. Many had experience of assessing and recording performance, but not using the methods or rating scales used here, although most had been raters in another study on one previous occasion (see Video observations).

### Video Observations

All observations were performed at the Defense Animal Center (DAC) (Leicestershire, UK), in three sessions (April, May and July 2013). Subjects attended in groups of between 1 and 11 participants. Each session lasted ~3 h, with two breaks in each as close to an hour apart as possible without disrupting the task. All but three (two HAS, one VS) of the observers had taken part in a previous rating experiment (31) using the same behavioral traits. For 11 subjects this had been the day before, for 11 observers it had been between 5 and 12 weeks previously, and for 2 VS observers, 2 h previously. The first task gave some experience of rating the behavioral traits, but the video clips were longer (~6 min) and observers were required to rate all of the traits at once. They had received the same definitions of the traits as in the pre-benchmark condition here, without the detailed descriptions of each performance level, so it was assumed that this would not affect the question of whether the descriptors were effective. As the raters who had taken part the previous task had been briefed on the purpose of rating performance and on common errors to try to avoid (e.g., halo), the three new subjects underwent a similar briefing. The instructions below were also reiterated to all participants before observations began.

When rating performance subjects were urged to:

a) assess each performance trait in isolation;
b) avoid being affected by an overall good or bad impression (halo effect), or being overly influenced by individual events;
c) avoid being influenced by any prior knowledge they had of the dog;
d) use the whole 1–5 scale whenever possible (e.g., avoid using just middle ranges);
e) assess the performance of the dog (not handler) in the particular search shown (not prior knowledge);
f) watch the whole clip before scoring any behaviors and assess performance based on the entire clip;
g) score the videos in silence to avoid influencing each other's scores.

Subjects were shown 10 videos of ~30 s in duration for each of 11 performance measures, starting with Control (for order of presentation see Table 1) and moving sequentially through to Detect & Locate. When each clip ended, the observers were asked to write their score for the particular performance trait on a recording sheet and this was repeated until five videos were rated. Subjects were then handed a list of benchmarks, or anchored terms, describing the 1–5 levels of that particular trait and asked to rate a further five videos with the anchored benchmarks to aid them. Benchmarks had been derived by the authors after watching and discussing the range of performance for each trait. They were deliberately kept as short adjective based sentences expanding on the original basic (one/two word) anchors. For example: Distraction, from [1] Very low - not distracted at all, through to [5] Very high - highly distracted; Motivation, from [1] Very low - no enthusiasm to search, to [5] Very high - very enthusiastic to search. After each trait had been scored, subjects were encouraged to discuss within the group how easy or difficult they found using the benchmarks and whether they felt that the benchmarks correctly described the different levels of performance. Due to time limitations of using expert handlers, all subjects watched the videos in the same order. We did not randomize or balance the order of the two conditions (bench-marked or not), as we anticipated there would be strong carry over effects after benchmarks were introduced.

### Statistical Methods

Analyses were performed for each trait within each classification (IBM SPSS Statistics 21), to answer the following questions:

1) **Does providing benchmarks increase rater reliability?**
We expected between-rater agreement, or reliability, to increase when subjects had the benchmarks for reference, as their idea of what constitutes the different levels should become more similar. We tested this by visual comparison of intra-class correlation coefficients (ICCs, two-way random effects with absolute agreement) in pre- and post-benchmark conditions. Average measure ICCs indicate how reliably a group of raters rated each of the traits (48), but cannot be generalized to indicate how well a single rater would perform. We therefore used single-rater ICCs, although average rater values are included to allow comparison with previous studies [e.g., (31)]. Reliabilities of > 0.7 were taken to indicate strong agreement.
2) **Does providing benchmarks change the range of ratings?**
  - Range restriction. If raters were using a greater range of the 1–5 scale (less range restriction) the spread of scores around the mean (standard deviation) will increase post-benchmark, as indicated by a significant change in standard deviation (SD) from pre- to post- benchmarking conditions (paired *t*-tests).
  - Mean scores. Univariate GLM, with pre/post benchmarks as the fixed factor and rater ID as a random factor, were used to test for a change in mean scores in pre- and post-benchmark conditions. As the videos were balanced across conditions the mean should “~3” for each trait in both pre and post conditions, but mean ratings might change as raters adjust their perception of the 1–5 categories within each behavior. If the raters were restricting ratings to a particular part of the scale then we would expect the mean score to change in the post-benchmarked condition; for example, if benchmarking reduces net acquiescence (use of mid to higher end of scale), mean scores should decrease.

## Results

Overall, average rater reliabilities were very high for both HAS and VS ratings; indicating good agreement amongst the group of raters (Table 2). Single rater ICCs were above the 0.7 threshold for strong reliability for 7 VS traits and 9 HAS traits, indicating that we could expect individual raters to produce reliable ratings. The exceptions to this were Independence and Speed in both classifications, as well-Stamina and Detect & locate for VS.

**Table 2:** Single rater agreement (ICC) between 11 raters, with 95% confidence intervals (upper and lower bounds) and average rater ICC for comparison.

**VS *N* = 17**	**ICC**	**Control**	**Motivation**	**Distraction**	**Search pattern**	**Stamina**	**Indication**	**Confidence**	**Thoroughness**	**Independence**	**Speed**	**Detect & locate**
Pre-benchmarks	Single value	0.930	0.744	0.837	0.755	0.690	0.900	0.722	0.719	0.561	0.662	0.597
	Lower bound	0.817	0.484	0.628	0.500	0.416	0.748	0.452	0.452	0.280	0.384	0.315
	Upper bound	0.991	0.961	0.977	0.963	0.950	0.987	0.957	0.956	0.918	0.944	0.928
	Average value	0.995	0.979	0.988	0.980	0.973	0.993	0.976	0.976	0.953	0.969	0.960
Post-benchmarks	Single value	0.877	0.892	0.633	0.782	0.764	0.854	0.850	0.554	0.826	0.780	0.772
	Lower bound	0.702	0.733	0.353	0.539	0.512	0.657	0.651	0.277	0.609	0.534	0.525
	Upper bound	0.984	0.986	0.937	0.968	0.965	0.980	0.980	0.915	0.976	0.968	0.966
	Average value	0.991	0.992	0.965	0.983	0.981	0.989	0.898	0.952	0.987	0.983	0.982
**HAS** ***N*** **=** **11**	**ICC**	**Control**	**Motivation**	**Distraction**	**Search pattern**	**Stamina**	**Indication**	**Confidence**	**Thoroughness**	**Independence**	**Speed**	**Detect & locate**
Pre- benchmarks	Single value	0.843	0.706	0.874	0.912	0.770	0.797	0.826	0.922	0.385	0.687	0.857
	Lower bound	0.629	0.422	0.686	0.768	0.503	0.550	0.596	0.789	0.129	0.390	0.651
	Upper bound	0.979	0.954	0.983	0.989	0.966	0.971	0.976	0.990	0.620	0.950	0.985
	Average value	0.983	0.964	0.987	0.991	0.974	0.977	0.981	0.992	0.873	0.960	0.985
Post-benchmarks	Single value	0.823	0.912	0.823	0.786	0.779	0.852	0.682	0.911	0.629	0.837	0.884
	Lower bound	0.592	0.768	0.590	0.532	0.522	0.645	0.392	0.767	0.327	0.618	0.708
	Upper bound	0.975	0.989	0.975	0.969	0.968	0.980	0.949	0.989	0.937	0.978	0.985
	Average value	0.981	0.991	0.975	0.981	0.959	0.949	0.976	0.991	0.983	0.988	0.984

*Highlighting indicates an increase (darker shading) or decrease (paler) in ICC of at least 0.1 in the post-benchmark condition. All ICCs were significant at P < 0.001*.

### Does Providing Benchmarks Increase Rater Reliability?

In the post-benchmarking condition, four VS traits improved noticeably in agreement (Motivation, Confidence, Independence, and Detect & Locate; Table 2), but three had lower levels of agreement (Distraction, Thoroughness and Speed). Distraction and Thoroughness did not reach 0.7 after adding benchmarks, despite both exceeding this threshold in the pre-benchmark condition. There were small positive changes in Stamina (enough to bring it over the 0.7 threshold for strong reliability) and Search Pattern, and similar changes - in the opposite direction - for Control and Indication.

The reliability of four HAS traits noticeably improved post-benchmarks (Motivation, Independence, Speed and Indication), whereas three had lower agreement (Distraction, Confidence and Search Pattern). Although Confidence and Independence did not reach 0.7 with the addition of benchmarks, the latter improved considerably (from 0.385 to 0.629). There were negligible changes in reliability for Control and Thoroughness (decreased), and Stamina and Detect & Locate (increased).

### Does Providing Benchmarks Change the Range of Ratings Used?

There was a significantly greater spread of scores around the mean (standard deviation) for Motivation in the post-benchmark condition, but lower spread for Control and Confidence and a tendency for Distraction (Table 3). With benchmarks, the VS observers rated Confidence, Independence and Thoroughness higher and tended to also rate Indication higher; whereas, they rated Distraction (and tended to rate Stamina) as lower. There were significant effects of rater identity on ratings for Stamina (*p* = 0.011), Confidence (*p* = 0.033), and Speed (*p* = 0.012).

**Table 3:** Difference in mean scores and standard deviation (within observer) between pre- and post-benchmark conditions for each performance trait (Univariate GLM, paired *t*-test); where the difference is significant the higher mean value is shown in bold and trends^t^ (also in italics).

**VS**	**Difference between mean scores**				**Difference in standard deviation**			
**Behavior**	**Mean pre- benchmarks**	**Mean post- benchmarks**	***F*-statistic**	**Significance**	**SD pre- benchmarks**	**SD post- benchmarks**	***t*-statistic**	**Significance**
Control	2.713	2.763	0.556	0.468	**1.701**	1.470	2.780	0.014
Motivation	2.930	2.912	0.072	0.791	1.407	**1.587**	−2.40	0.030
Distraction	**3.550**	3.163	5.333	0.036	*1.654^***t***^*	1.418	2.113	0.052
Search pattern	2.838	2.900	3.021	0.103	1.413	1.441	−0.227	0.823
Stamina	*3.275^***t***^*	3.088	4.494	0.051	1.339	1.236	1.139	0.273
Indication	3.075	*3.275^***t***^*	3.750	0.072	1.585	1.433	1.637	0.122
Confidence	2.988	**3.363**	32.767	<0.001	**1.843**	1.453	4.543	<0.001
Thoroughness	2.825	**3.362**	16.44	0.001	1.351	1.303	0.390	0.702
Independence	3.225	**3.613**	13.874	0.002	1.266	1.408	−1.420	0.176
Speed	3.212	3.206	0.004	0.952	1.271	1.203	0.632	0.537
Detect & locate	2.863	2.913	0.128	0.725	1.552	1.492	0.777	0.449
**HAS**	**Difference between mean scores**				**Difference in standard deviation**			
**Behavior**	**Mean pre- benchmarks**	**Mean post- benchmarks**	***F*****-statistic**	**Significance**	**SD pre- benchmarks**	**SD post- benchmarks**	***t*****-statistic**	**Significance**
Control	2.545	2.655	0.803	0.391	1.506	1.460	0.571	0.581
Motivation	2.982	2.982	0.000	1.000	1.387	**1.560**	−2.893	0.016
Distraction	**3.491**	3.055	20.426	0.001	*1.633 ^***t***^*	1.495	2.029	0.070
Search pattern	2.964	**3.218**	12.564	0.005	1.625	1.376	1.874	0.090
Stamina	**3.618**	3.073	14.063	0.004	1.068	*1.300 ^***t***^*	−2.144	0.058
Indication	2.636	**3.309**	190.139	<0.001	1.380	1.421	−0.247	0.810
Confidence	2.945	**3.405**	7.183	0.023	1.440	1.476	−0.492	0.633
Thoroughness	2.691	2.782	2.119	0.176	1.670	1.640	0.368	0.720
Independence	2.782	2.927	0.907	0.363	1.189	1.29	0.649	0.531
Speed	2.564	**3.382**	21.182	0.001	1.101	1.100	0.019	0.985
Detect & locate	2.873	**3.291**	9.446	0.012	1.548	1.509	0.388	0.706

For HAS raters, the only behavior where there was a significant difference in the spread of scores around the mean (standard deviation) was Motivation, where observers used a wider range of values in the post-benchmark condition. There was a trend in the same direction for Stamina, but in the opposite direction for Distraction, with observers tending to use a narrower range of scores when benchmarks were included. They rated Stamina and Distraction lower with benchmarks, and rated Confidence, Search Pattern, Speed, Detect & Locate, and Indication higher. There were significant effects of rater identity on Motivation (*p* = 0.014), Distraction (*p* = 0.010), Search Pattern (*p* = 0.036), Indication (*p* < 0.001), which with the exception of Confidence and Independence, coincides with the behaviors showing the greatest change in ICC.

## Discussion

In the pre-benchmarking condition average rater reliabilities were generally very high (>0.7) for both classifications, with single rater ICCs above the 0.7 threshold for strong reliability for most behavioral traits. This means that an individual rater within the group is likely to show good reliability in their ratings. The exceptions to this were Independence and Speed in both classifications, and for VS, ratings for Stamina (although this was very close to the threshold at 0.69) and Detect & Locate. Using benchmarks has the potential to alter how the handlers rated several of the traits, as evidenced by changes in rater agreement and in differences between scores. Motivation for example, showed an increase in reliability amongst raters of both VS and HAS classifications when benchmarks were provided, and the spread of scores increased in the post-benchmarking condition without any change in mean scores, suggesting that as well as bringing raters together in their understanding of the trait, they were also better able to use the full range of the scale. Thus, for Motivation the use of benchmarks achieved the initial aims. However, the benefit of benchmarking was not universal, with the size and direction of effects varying between the VS and HAS groups and according to the behavioral trait being rated.

### Does Providing Benchmarks Increase Rater Reliability?

Improvements in reliability occurred when benchmarks were provided for Motivation and Independence (HAS and VS), Speed and Indication (HAS only), and Confidence and Detect & Locate (VS only). As agreement was higher, the benchmarks appeared to bring the raters' interpretation of category meanings for these traits closer together. It seems logical that the relative improvement in the post-benchmark condition should be greater for traits that may be conceptually harder to rate, such as those that are more abstract and less easily quantifiable [see (31)]. For example, raters are likely to hold clearer a-priori representations of the difference between a score 3 and a score 4 for Control, a trait with high observability (49), where we expect them to already have a concept of differing levels of dogs' responsiveness to commands, compared to traits such as Independence and Motivation, which are conceptually more abstract or more evaluative (49). This was the case for these behavior traits: reliability for both Motivation and Independence improved with benchmarks for both dog classifications, whereas for Control ICCs changed very little and in fact, declined very slightly.

Whilst we may have expected any change in the reliability of less evaluative traits (e.g., Control) to be of smaller magnitude compared to traits where there was greater room for improvement, it is not immediately clear why benchmarks had no impact at all on traits such as Control (HAS and VS); or why agreement for Distraction (HAS and VS), Confidence and Search Pattern (HAS), and Thoroughness and Speed (VS), decreased. One possibility is that where there was negligible change in reliabilities, rater conceptions of the trait levels may have already matched the provided benchmarks, hence leading to no improvement. Alternatively, the lack of change or decrease in agreement for some behaviors could indicate a reluctance of some observers to change their a-priori assumptions (non-compliance) about what constitutes each level of performance even with the benchmarks in front of them. This could, in fact, prove to be a particular issue with very quantifiable traits, where raters might hold steadfast ideas of performance, and especially in this group of raters which included many with considerable experience and expertise, whilst less experienced raters may show differential effects. It is also possible that our adjective-based descriptors were insufficient to make a difference to these ratings. This requires further investigation.

There are also limitations to the study, in that the design was inevitably unbalanced, with the post-benchmarking condition having to come after the pre-benchmarking. This design was deliberately selected to avoid carry-over and memory effects, however a consequence is that some of the changes post-benchmarking may have resulted from raters having more practice with scoring. This could have been particularly important for those raters with less experience, who may not have seen many examples of dogs performing at the very poor end of the spectrum previously. Although the lack of any universal increase in agreement suggests that any “practice” effects were limited.

### Were Reliabilities Comparable to Previous Studies?

Both average and single measures agreement was generally high, and higher than the author's previous studies using the same traits (24, 31), and variability between raters was lower. Even without benchmarking, the predicted ability of a single rater to reliably score performance was within the levels of acceptability (>0.7) for most traits in this task, unlike in Rooney and Clark (31). This is considerably higher than most estimates of inter-rater agreement when rating aspects of dog behavior [e.g., see (50); see (25)], but similar to that found by Fratkin et al. (14) when rating search dog performance. Raters may have been better because, in this task the vast majority had previous experience of using the ratings; although as there seemed to be no universal benefit from practicing ratings between the pre- and post-benchmarked conditions (see above), the more likely explanation is that the task itself was inherently easier. Raters were focusing on one trait at a time here, rather than trying to remember multiple traits at once, and perhaps more importantly, here raters assessed behavior from very short clips designed to illustrate a particular level of performance, whereas in Rooney and Clark (31) they had to make an assessment based on assimilating 6 min of behavior during which time performance level could fluctuate. They may also be artificially inflated as the videos used in this study were selected to reflect the full range of each of the rating scales. This was deliberate as the same clips were to be used to train personnel how to rate searches using the full extent of the scale, however it is likely easier for judges to distinguish between behaviors that vary greatly in magnitude than those that are close to a midpoint, and normally encountered. Caution should therefore be exercised when extrapolating the results of this study to actual performance in the field, as the methodology in Rooney and Clark (31) is a closer representation to the actual task facing handlers. This raises an important point when developing measurement tools encompassing rating scales for several aspects of performance, as although raters may be reliable at scoring individual traits in an experimental setting, this may not equate to ability in the field. Additionally we further need to test the value of benchmarks when rating numerous traits simultaneously.

### Does Providing Benchmarks Change the Range of Ratings Used?

The addition of benchmarking either did not affect the mean score, or lead to an increase, so that in the post-benchmarking condition most traits were scored higher than “3” on average (as opposed to lower than 3); which was more evident in HAS ratings. The opposite was true for Distraction, but unlike the other traits, low scores for this trait are more positively valenced (no or low Distraction is ideal). This suggests that some raters became more lenient when provided with benchmarks.

The quality of ratings and the degree of rater error are a reflection of the measurement tool, the rater, and the interaction between the two. Although our treatment altered the way that the measurement tool was presented to raters, it is likely that the impact of this change will vary between raters based on characteristics inherent to the individual. Previous studies have demonstrated that observers differ in their ability to rate traits (24, 31); therefore, it also seems likely that they will differ in their ability to effectively use benchmarks, and here we did detect several main effects of rater identity. Differences may simply be due to factors such as differing levels of experience of working with search dogs and assessing performance. However, the psychology literature commonly reports the occurrence of raters resorting to particular styles of responding [e.g., (51)], which can be pervasive, despite instruction on avoiding bias (52). Such response styles include scoring only within mid-to-positive range values (net acquiescence) and responding only at the extremes of the scale (8, 32).

While benchmarking might make all points on the scale equally salient and accessible, thus potentially reducing rater biases, it could also increase response style bias in some people. For example, negative extremes become more salient in benchmarked scales compared to when categories are unlabelled, which may lead to greater net acquiescence (41). Although our scales were labeled based purely on the amount of a particular trait rather than being explicitly positively or negatively valenced (i.e., good, poor etc.), dog handlers have shown leniency bias in ratings using these scales (24), and it is likely that they will naturally associate favorable and negative connotations with particular ends of the scale. For example, VS observers used a narrower range of scores for Control and Confidence when presented with benchmarks, with scores for the latter also being higher post-benchmarking. So for these traits, benchmarking appeared to cause some raters to be more reluctant to use scale extremes; and for Confidence, this was also associated with more positive ratings, which could be attributed to greater net acquiescence. Therefore, we would recommend that benchmarks cannot be universally applied with global benefit. The next step should be to try to understand changes as a result of benchmarking at the individual rater level and between classifications (e.g., comparing why HAS and VS observers differed).

### Further Development to Increase Effectiveness of Benchmarks

The lack of improvement or decrease in agreement for some traits may also have been the result of deficiencies in those specific benchmarks used, leading to greater uncertainty in trait meanings or greater non-compliance in using them by observers who disagreed with the descriptions provided. Some benchmarks were perhaps not detailed enough, or too “generic.” Our benchmarks were adjective based (e.g., Very, Low, Intermediate, High, and Very High) and although a “descriptive” statement followed, this was intentionally short (to allow inclusion in an operational data recording instrument) and in most cases also adjective-based (e.g., rarely, often, sometimes, usually, always). They may therefore have had limited benefit in helping raters to categorize behaviors and were open to subjective interpretation, e.g., when discussed during the session, raters felt that the benchmarks for Detect & Locate needed some alteration. A further point to consider is that ratings may also change over time, e.g., if the quality of dogs drifts, and it is therefore important to have quality control in place such as the video based training resource developed here which could be used to standard set and ensure temporal standardization in ratings.

In general, feedback from observers on the value of the benchmarks was positive: raters generally preferred scales with benchmarks; hence they may prove valuable at increasing compliance even if the scale use improvement is variable or unproven. We suggest that including more descriptive terminology in the benchmarks may be valuable. Descriptive statements elaborating on examples of the precise behaviors that constitute particular levels of performance have been found to increase reliability in ratings more than purely adjective-based benchmarks (37). The development of descriptive benchmarks is more time consuming than simple adjective-based terminology and care needs to be taken to ensure the validity of the scale [see (53)]: e.g., in scales intended to be balanced, that the scale midpoint equates to this conceptual performance midpoint (39, 53). Care must also be taken not to make the descriptions too lengthy as to increase rater cognitive burden and subsequent non-compliance or careless rating [e.g., (54)]. Descriptive benchmarks need to be derived using feedback from and discussion with raters to ensure the distinction between levels is meaningful and acceptable to the rating population. This could be achieved through an iterative process involving consultation with the end-users. For example, we could add a further step to examine whether benchmarked levels match rater perceptions, by asking raters to describe the different levels of performance in their own words. If levels are found to differ then the benchmarks should either be altered to match the observer levels (if these are in fact deemed to be the correct categorisations), or alternative methods employed to persuade and train raters to use the benchmarked levels.

### Rater Training

A further and potentially valuable approach to improving reliability, in conjunction with benchmarking, is rater training. Simply providing the list of benchmarks was insufficient for at least some of the raters, who may benefit from being able to discuss and see examples of the difference between performance levels. Evaluative accuracy training focuses on increasing validity by moving respondent ratings closer to a reference or gold standard, through dimension-relevant judgements (33). Instead of the handlers comparing their internal categorization with a written list of benchmarks, their internal representation of the different levels of behaviors is altered to match the desired levels, making the process of categorizing behaviors using the new shared framework automatic and internal. Frame-of-reference (FOR) training is widely used in other disciplines and teaches raters to use a common conceptualization (or frame of reference) when observing and evaluating performance (38) providing gold-standard examples of the different levels has been proven to improve rater accuracy in many different scenarios (55–57), particularly when combined with anchored rating scales (38).

### Limitations to the Methodology

The practical constraints of working with a time-limited operational cohort of raters meant that it was impossible to counterbalance the experimental design and randomize the order of presentation of videos. Therefore, all participants watched the searches in the same order, and for each trait the same examples were benchmarked. Efforts were made to balance the two sets of video based on previous ratings by handlers (31) and the authors' assessments. However, it remains possible that there were inherent differences between the sets of videos selected, which led to some of the detected differences between conditions. The sample sizes of both the number of videos observed per trait and the number of participants were relatively small. The results show traits displaying differential effects of benchmarking, which could in theory be attributed to initial differences between videos. In the absence of a control group, it is impossible to draw strong conclusions regarding the effect of benchmarking. We therefore conclude that similar to the way in which codings of behaviors have been demonstrated to lack the often presumed value relative to more subjective ratings [e.g., (27, 58)], the addition of adjective based descriptors here did not demonstrate clear universal value to rating scales.

There may be value in further investigating these concepts using larger groups of observers. Here, we did not have adequate sample sizes to explore the impact of rater experience and it may be that this will impact rater's ability to reliably use rating scales based on adjectives. Although, as discussed, pre-conceived ideas may also make experienced handlers less receptive to using the scales. Using researchers as well as trained dog handlers could facilitate obtaining a larger sample size as it has been demonstrated that their ability to rate searches is comparable to expert dog handlers (14, 28). This could enable a replication of this study with balanced presentation of benchmarked and unbenchmarked scales, randomization of videos and potentially examine the effect of experience rating dogs in using the scales. It would, however, not be possible to use the realistic operational training searches from the field used in this study as these are only viewable by military personnel. Therefore, despite the limitations in design, this study provides a rare opportunity to measure the application of rating scales in a realistic military environment.

## Conclusions

Rating scales with and without benchmarks, are widely used in human and animal sciences, yet variable levels of consideration are given to how aspects of the design of the scale might alter the validity of the results obtained. In Rooney and Clark (31) we illustrated the importance of looking beyond overall correlations between behaviors when assessing scale validity and here we demonstrate that even relatively small additions to the amount of information given to raters can have important consequences for the data obtained. This study illustrates that to produce optimal performance measures, it is important to validate all aspects of design of the measurement tool and consider the amount and type of information provided to raters, as this can have both positive and negative impacts on ratings. The changes seen here were equivocal, but the feedback received from subjects suggests that handlers can benefit from additional information when scoring, especially for certain traits where providing benchmarks may potentially reduce ambivalence in scoring, ambiguity of meanings, and cognitive difficulty. However, benchmarking was not demonstrated to be universally valuable, and simply providing very basic adjective-based anchors may result in limited overall improvement and potentially more disagreement for some terms. Before performance measurement tools, such as the example developed here for the working dog community, are used, we recommend iterative development of benchmarks, given in conjunction with training such as Frame Of Reference, whereby raters can view and discuss differing levels of performance. This is likely to be the most effective method of improving rater reliability, by training those inexperienced in assessing performance as well as altering any pre-conceived ideas and bringing all raters closer to common conceptualization of the meaning of traits.

## Data Availability Statement

The datasets presented in this article are not readily available because they contain information on military working dog performance and are thus sensitive. Requests to access the datasets should be directed to Nicola J. Rooney, nicola.rooney@bristol.ac.uk.

## Ethics Statement

The studies involving human participants were reviewed and approved by Faculty of Medical & Veterinary Sciences (FMVS) Research Ethics Committee. Written informed consent for participation was not required for this study in accordance with the national legislation and the institutional requirements. The animal study was reviewed and approved by Animal Welfare & Ethical Review Board. Written informed consent was obtained from the owners for the participation of their animals in this study.

## Author Contributions

CC and NR: conception of idea, methods development, and preparation of manuscript. CC: majority of data collection and analysis.

## Conflict of Interest

The authors declare that the research was conducted in the absence of any commercial or financial relationships that could be construed as a potential conflict of interest.
